# Functional Tests Predicting Return to Work of Workers with Non-Specific Low Back Pain: Are There Any Validated and Usable Functional Tests for Occupational Health Services in Everyday Practice? A Systematic Review

**DOI:** 10.3390/ijerph20065188

**Published:** 2023-03-15

**Authors:** Heikki Hurri, Toni Vänni, Elli Muttonen, Fabrizio Russo, Sergio Iavicoli, Leena Ristolainen

**Affiliations:** 1 Research Institute Orton, Tenholantie 10, 00280 Helsinki, Finland; 2 Faculty of Medicine, University of Helsinki, PL 63, 00014 Helsinki, Finland; 3 Terveystalo, Jaakonkatu 3, 00100 Helsinki, Finland; 4 Department of Orthopaedic and Trauma Surgery, Campus Bio-Medico University of Rome, Via Alvaro del Portillo 200, 00128 Rome, Italy; 5 General for European and International Relations, Ministry of Health, General for Communication and International Affairs, Ministero della Salute, 00144 Roma, Italy

**Keywords:** low back pain, work, return to work, functional tests

## Abstract

The literature predominantly advocates subjective perception of disability and pain as an outcome measure for the functional evaluation of patients with low back pain (LBP). Physical outcome measurements are almost completely ignored. In this systematic review, we focused on physical functional measurements that can contribute to the prediction of patients’ return to work (RTW) readiness after sick leave or rehabilitation. Searches were conducted in July 2022 without any time limit in the Cochrane Library, PEDro, PubMed and Scopus databases for functional and clinical tests reliable and applicable in clinical practice without demanding equipment. Two independent researchers extracted the data from the included articles in a standardised data collection form, and a third researcher validated the data extraction. No date restriction was applied. We followed the Preferred Reporting Items for Systematic Reviews and Meta-Analyses (PRISMA) guidelines in conducting the review. We found seven original articles, including six with an impact on predicting RTW. We found four fair and three poor original studies fulfilling our criteria. We found the Back Performance Scale (BPS) and back endurance test to be the most promising tests for occupational health service and the clinical practitioner. Radiation of back pain, with or without neurological deficiencies, had some predictive value in terms of RTW, too. The working conditions vary a lot, which causes inconsistency in the studies and in their interpretation. Functional tests could complete the widely used working ability evaluations methods such as the Work Ability Index (WAI) and are worth considering for future research. Overall, more research is needed in this field. The question of when LBP patients can resume everyday activities and work is not possible to determine with functional tests alone. Psychosocial aspects and work demands must be considered. PROSPERO: CRD42022353955. The study was funded by the University of Helsinki.

## 1. Introduction

An estimated 7.5% of the global population are affected by LBP either acutely or chronically, making LBP a major burden both individually as well as socioeconomically [1,2,3]. Up to 90% of all LBP cases are considered “non-specific” [4], i.e., the cause of pain is not known [5], and pain can come from any part of the spine supplied by pain nerves. While most patients heal by themselves or with conservative treatment, 5–20% develop a chronic condition with an increased prevalence among the older population [6]. Chronic low back pain (CBLP) is defined as pain lasting longer than 3 months [7]. The functional evaluation of patients with unspecific subacute or chronic low back pain (LBP) is a continuous challenge, particularly in occupational health service.

The prevailing literature advocates multifaceted follow-up evaluations for patients with LBP. According to a comprehensive review by Chapman et al. [8], the most important domains are pain, function and quality of life. For pain, they recommended the Visual Analogue Scale (VAS) and the Numeric Pain Rating Scale (NPRS) because of their ease of administration and responsiveness. For function, they recommended the Oswestry Disability Index (ODI) [9] and the Roland Morris Disability Questionnaire (RMDQ) [10]. Notably, functional tests were not mentioned. The same observation was made in the review by Froud et al. [11], namely, that functional measurements are neglected as outcome measures in studies of patients with LBP.

This is contradictory to the principles of the International Classification of Functioning (ICF) [12], which combines bodily functions and the social consequences of diseases and advocates a holistic view when evaluating the outcome of diseases. In addition to the well-grounded conceptual theory of the ICF, functional tests for patients’ back pain are used and required in occupational health service. This presents a dilemma: no functional test for patients with LBP has so far succeeded in gaining unanimous acceptance as the method of choice to predict return to work (RTW). A good test should be valid, reliable and responsive, and predict RTW or illustrate the patient’s working capacity.

The demand for practical functional tests for patients with LBP is obvious. Modern occupational health service aims to contribute to the total management of working ability within the employing company. Modified work and alternative work or duties [13,14] are increasingly adopted as a tool to combat total working incapacity. Physical tests, i.e., measurements, are sounder bases in the decision making when different work measures are planned. The same argument applies for social insurance institutions which prefer functional tests or other clinical evidence to mere subjective perception. This calls for the development of tools to predict the RTW of patients with LBP.

Work demands vary hugely, so it is almost impossible to find a functional test that could give a comprehensive view of any patient’s working capacity to RTW after an episode of back pain. However, we aimed to search the literature to find the best available and suitable functional tests for patients with LBP (i.e., subacute or chronic unspecific low back pain) to predict their RTW, knowing that any functional test should be accompanied by psychometric and social evaluations. We focused on clinical and functional tests that are applicable in everyday practice without any special or expensive technology and that can be conducted within 20 min visits. The physical test must be reliable enough to be usable and it also has to predict to some extent the RTW. Accordingly, we omitted demanding functional tests planned for special situations, such as tests for fire-fighters that use isokinetic measurements [15], and tests whose reliability is not tested. Definitions of terms are shown in Table 1.

## 2. Materials and Methods

This review was registered at the International Prospective Register of Systematic Reviews: PROSPERO (CRD42022353955). We followed the Preferred Reporting Items for Systematic Reviews and Meta-Analyses (PRISMA) guidelines in conducting the review and reporting our findings [22].

### 2.1. Data Sources and Search Strategy

A comprehensive literature search was conducted in July 2022 in the Cochrane Library, PEDro, PubMed and Scopus databases, combining the following keywords: ((low back pain AND clinical test AND return to work) OR (low back pain AND clinical test AND disability pension) OR (low back pain AND clinical test AND sick leave) OR (work related low back-pain AND clinical test) OR (work related low back-pain AND physical test)). No language restrictions were applied, nor was any date limitation. Searches were also conducted for previous systematic reviews and cross-references.

### 2.2. Inclusion and Exclusion Criteria

The inclusion criteria were as follows: (1) studies: original articles; (2) participants: working patients with chronic LBP (lasting more than 3 months); (3) interventions: objective tests to measure patients’ physical performance; and (4) outcome measures: the primary outcome measure was work ability, and the outcome indicator was RTW after sick leave. The exclusion criteria were as follows: (1) studies in which the subject had an acute cause of LBP, including fracture, osteoporosis and malignancy; and (2) letters, conferences and commentaries.

### 2.3. Data Extraction

For all the included articles, the following data were extracted: (a) study characteristics and study design (author, year and sample size); (b) participants (sex, age, number of participants and inclusion and exclusion criteria); (c) intervention and comparison groups; (d) follow-up time; (e) functional tests; (f) outcome measures; (g) results; and (h) conclusions.

Due to the limited number of included studies, a quantitative analysis was deemed inapplicable to this study.

### 2.4. Methodological Quality Evaluation and Risk of Bias (RoB)

Two independent researchers (HH and LR) extracted the data from the included articles in a standardised data collection form, and a third researcher (TV) validated the data extraction. Any disagreements between the researchers were resolved by a third researcher (TV) and consensus was attained. Quality assessments were evaluated with the Study Quality Assessment Tool criteria developed by the National Heart, Lung and Blood Institute [23]. The quality of the studies was assessed as good, fair or poor.

We assessed risk of bias (RoB) of the studies according to the principles presented by Furlan et al. [24]. The bias was assessed in a structured and fixed set of domains, focusing on design of the study, conduct, generalisability of the results and reporting. Two review authors (HH and LR) independently assessed RoB. We used a consensus method to resolve disagreements and consulted the third review author if disagreements persisted. We scored the criteria as “high risk” or “low risk”. “High risk” for a study was achieved if at least one domain was of high RoB or there were some concerns for multiple domains that substantially lowered the confidence in the result. In cases of “high risk” evaluation of RoB, the overall quality evaluation was lowered from good to fair and from fair to poor.

## 3. Results

### Literature Search Results

**Figure 1:**
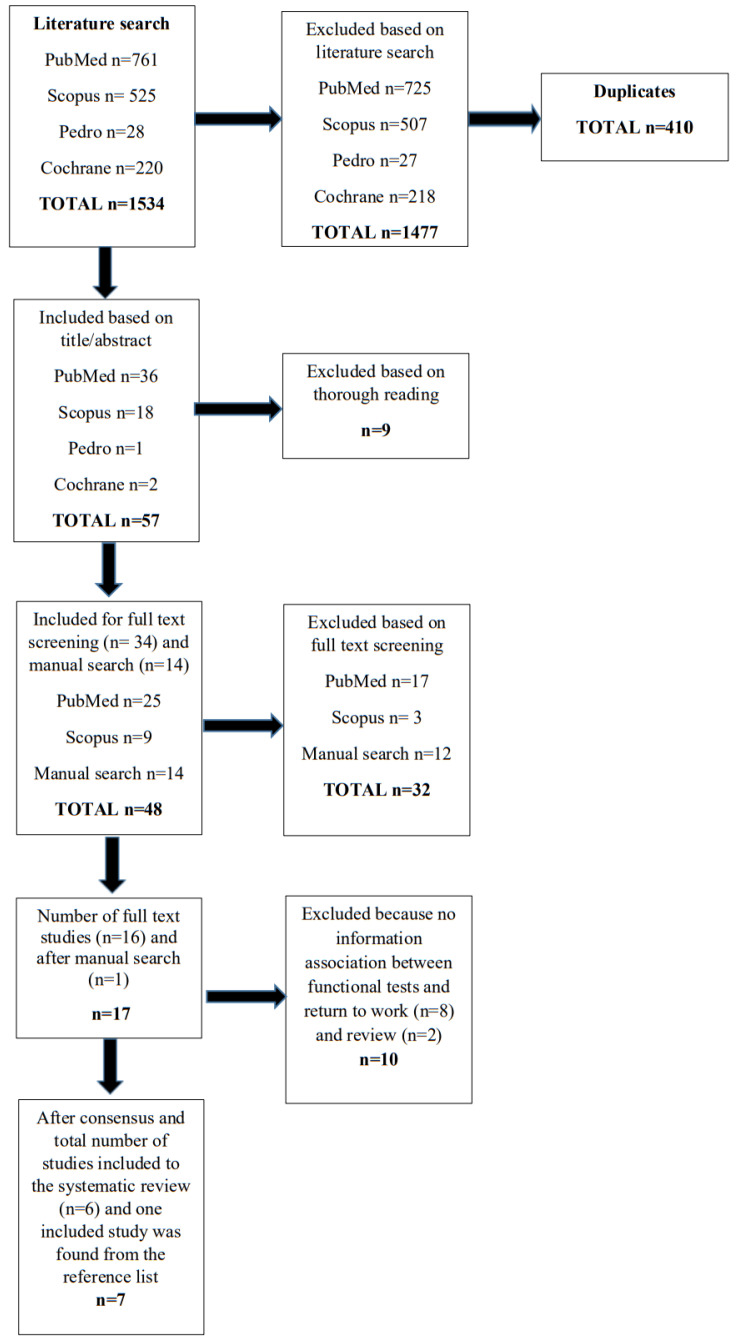
Flowchart of the search history.

Our search yielded 1534 records according to the predefined search strategy, of which 410 records were duplicates. A total of 1067 studies were excluded after screening the abstract. The full text of 48 articles was retrieved for detailed evaluation. Finally, we identified 17 original articles to consider in our systematic review. After a detailed review, two of the authors (HH and LR) included seven articles in the final quality study assessment. Eight articles were excluded because there was no information about the association between functional tests and RTW [25,26,27,28,29,30,31,32], and two review articles [8,33]. The final literature review yielded seven articles fulfilling the criteria. One article was found through manual research, but the others were found through screening the databases. The flow chart is presented in Figure 1. Baseline details of the included articles are presented in Table 2.

The outcome measures, results and conclusions of the finally accepted articles are presented in Table 3. One article, by Christiansen et al. [34], fulfilled the research criteria but showed a negative result for a clinical phenomenon called centralisation as an indicator of RTW. The pain centralisation phenomenon is a term used in a form of physical therapy known as the McKenzie Method. Centralisation describes a phenomenon whereby pain in a leg or buttock suddenly shifts to a spot closer to the spine if the spine is moved or manipulated [35]. Christiansen et al. [34] studied this phenomenon in a sample of 351 patients on sick leave because of LBP with or without sciatica. The patients were classified into three groups according to their pain response: centralisation, peripheralisation or no response. At the one-year follow-up, 65% of the patients had returned to work. All the pain response groups showed significant and clinically important improvements in both pain and disability. No significant differences were found between the pain response groups in any outcome measure.

**Table 2:** Baseline details of the included articles.

Author, Year, [Reference Number], Country	Study Design	Participants			Interventions	Comparisons	Follow-Up	Functional Tests
		Patients	Inclusion Criteria	Exclusion Criteria				
Christiansen D et al., 2010 [34], Denmark	Prospective cohort study	331 patients Female n = 169/331 (51%)	* Partly or fully sick-listed for 4 to 12 weeks from work because of LBP * Age 16–60 years * Living in the municipality of some towns in Denmark	* Registered as unemployed * Serious spinal pathology * Progressive neural compression indication needs for surgery * Suspected progressive paresis or cauda equina syndrome * Low back surgery the preceeding year or previous lumbar fusion * Pregnancy * Dependency on drugs or alcohol * Primary psychiatric disease * Not able to speak and understand Danish	* Physiotherapy examination included a standardised mechanical evaluation * Three groups * Centralisation * Peripheralisation * No response	None	12 months	Low back examination * Evaluation of posture * Curvature of the spine * Range of motion * Neurological function * Laseque and femoral stretch test * Spring test * Tenderness with percussion * Standardized manual examination of tenderness of muscles
Strand LI et al., 2001 [36], Norway	A cohort study to examine the intertester reliability, validity, and responsiveness of the pick-up-test	Patients with back pain (n= 164), had been on long-term sick leave Female: Intertester 12/24 (50%) Back pain 93/164 (57%)	Intertester reliability (n = 24) Validity (n = 295) Responsiveness (n = 117)		The patients participated in a multidimensional outpatient rehabilitation programme.	None	12 months	Pick up piece of paper from the floor
Strand LI et al., 2002 [37], Norway	Randomized controlled trial	Patients with back pain (n = 249), included (n = 114) Female: 68/114 (60%)	Patients on long-term sick leave (>2 months, <1 year) because of musculoskeletal conditions	* Pregnancy, substance abuse, and illnesses such as progressive nervous system disease, serious cardiac disease, acute infection * Patients must have sufficient knowledge of the language	Tests in the baseline and at follow-up		12 months	Back Performance Scale (BPS) * Sock test * Pick-up test * Roll-up test * Fingertip-to-floor test * Lift test
Magnussen L et al., 2007 [38], Norway	Randomised controlled trial	Participants had received disability pension for more than one year (n = 89) Female 56/89 (63%)	All individuals on disability pension due to back pain * Under 56 years * Having received full disability pension payment for more than one year		Intervention group (education, motivation, vocational counselling), n = 45 Intervention: 2 sessions lasting for 3 h, 2 or 3 days apart, was organized in groups (5–11), included 2 h of lectures, 3 h of motivational interviewing, 1 h of information was provided from the social insurance office and work office. After the group sessions the participants were offered individual follow-up by a physician and a nurse and medical examination (29 accepted)	A control group (n = 44)	12 months	Back Performance Scale (BPS) * Sock test * Pick-up test * Roll-up test * Fingertip-to-floor test * Lift test
Bendix AF et al., 1998 [39], Denmark	A prospective clinical trial that involved six groups of patients with chronic low back pain (LBP) selected from a large cohort (n = 816)	816 patients with chronic low back disability during June 1991 and June 1995 Group 1 female n = 416/621 (67%) Group 2 female n = 357/534 (67%) Group 3 female n = 195/292 (67%) Group 4 female n = 134/195 (69%) Group 5 female n = 113/157 (72%) Group 6 female n = 82/109 (75%)	* Patients who had chronic disabling LBP for at least 6 months * A threatened job status (out of work because of back pain or at high risk of losing a job because of too many days of sick leave) * People who still had a job available but who were close to losing it because of their back problems. * Patients were 18 to 61 years	* Indication of actual surgical treatment * Not able to speak and understand Danish	Six groups were formed: GROUP 1: all patients—randomised or referred directly—undergoing the FR program (n = 621) GROUP 2: patients completing the FR program and participating in a 1-year follow-up (n = 534) GROUP 3: patients from Group 2 who were unable to work when entering the project, analysed separately with the purpose of testing prediction of the ability to work (n = 292) GROUP 4: all patients initially randomised to a control group of either no treatment or one of three possible outpatient programs (n = 195) GROUP 5: patients completing a control group program and participating in a 1-year follow-up (n = 157) GROUP 6: patients from Group 5, who were unable to work when entering the project (n = 109)	Several comparisons were made within and between the groups: * Do any pretreatment clinical variables predict which patients will or will not have returned to work 12 months after participation in FR program? * Is it possible, by recording pretreatment clinical variables, to predict which patients will withdraw from FR program? * Are there differences in predictive data between patients treated in FR program or in control groups? * Is there a correlation between pretreatment clinical variables and decrease or increase in pain and disability after 1 year in patients in the different groups? * Is there a correlation between pretreatment clinical variables and the patients ‘subjective overall assessment 12 months after participation in one of the groups?	12 months	Isometric abdominal muscle endurance and isometric back muscle endurance (time in seconds)
Kool J et al., 2002 [40], Switzerland	Longitudinal prospective cohort study	99 patients with chronic LBPFemale 15/99 (15%)	* To enable return to work (RTW) at follow-up * Age 20-60 years * Patients willing to go back to full time work were included * More than 6 weeks off work during the preceding 6 months of LBP * Sufficient understanding of language (German, Serbo-Croat, Spanish, Italian) to fill out the questionnaires	* Housewives and part-time employees * Comorbidity contributing to disability and sickness leave	Interdisciplinary rehabilitation: The average length of stay in the rehabilitation centre was 28.2 days. The treatment duration was 16.2 h/week, with an emphasis on active treatment such as strength and endurance training, exercise therapy, back school, and swimming. Psychological interventions, relaxation techniques and passive treatments. Purpose of the study: to evaluate the reliability and predictive validity of four prognostic tests and the psychosocial factors for non-RTW.	None	12 months	Only baseline data * Step test * Pseudo Strength test * Behavioural signs * Tenderness * Simulation tests * Distraction test * Regional disturbances * Overreaction
Loisel P et al., 2002 [41], Canada	Proscpective cohort study of 104 sick-listed back pain patients, who were randomised into four treatment groups: standard care (control), clinical–rehabilitation intervention, occupational intervention, and the Sherbrooke model (acombination of the clinical–rehabilitation and occupational interventions).	104 patients with back painFemale 42/104 (40%)	Workers with low back pain who had been absent from work for more than 4 weeks * Age 18–65 years * Thoracic or lumbar back pain had arisen at work	* Pregnant workers * Workers with spine fracture, significant degenerative spine disease, a nonmechanical spine disease, major comorbid condition that might limit participation	To assess the discriminative and predictive validity of the Quebec Task Force Classification (QTFC) during the subacute phase of disability Randomised into four treatment groups: * Standard care (control) * Clinical—rehabilitation intervention * Occupational intervention * Clinical—rehabilitation and occupational intervention Occupational intervention: included visits to the study occupational medicine physician and a participatory ergonomic intervention Clinical-rehabilitation intervention: clinical examination, back school, multidisciplinary work rehabilitation intervention	Standard care (n = 26)	12 months	QTFC: * Categories 1 to 3: based on the location of pain * Category 4: result of clinical examination * Category 5 to 7: results of imaging investigations * Category 8 to 10: response to treatment * Category 11: spine disorders seldom seen in occupational medicine Clinical tests: * Location of pain, posture, mobility of the spine and lower limbs, palpation, pain in the sacroiliac joint and piriformis muscle, and signs of nerve root tension and neurologic impairment

FR = Functional Restoration; LBP = low back pain; RTW = return to work; QTFC = Quebec Task Force Classification; BPS = Back Performance Scale.

Of the remaining six articles, three partly overlapped. Strand et al. [36] presented the Pick-up test and then, a year later [37], the Back Performance Scale (BPS) with five items: the Sock test, the Pick-up test, the Roll-up test, the Fingertip-to-floor test and the Lift test.

The latter is a further development of functional tests for patients with LBP after the Pick-up test. In Strand et al.’s study [36], the BPS sum scores discriminated between patients with different RTW statuses, and the BPS sum score was more predictive than the separate tests. In addition, the BPS was used in a randomised rehabilitation study by Magnussen et al. [38], but the number of participants completing the programme was low; only 29 of the participants (64%) in the intervention group completed the intervention. Twice as many in the intervention group (n = 10, 22%) had entered a RTW process with the controls (n = 5, 11%) [38]. Better physical performance was one of the factors related to RTW along with positive expectancy and less pain. The small number of the subjects hampers the conclusions of that study.

Back muscle endurance was connected to working capacity in a study by Bendix et al. [39]. In their study of a functional restoration programme, poor back muscle endurance was connected to receipt of a disability pension. RTW after the one-year follow-up was related to good pre-treatment back muscle endurance, and the relief of back pain in the functional restoration programme was related to pre-treatment back muscle endurance.

Kool et al. [40] conducted a prospective cohort study of 99 patients with chronic LBP. Upon entry to the study, physical workload, time off work, unemployment and nationality were recorded. The study investigated four tests with an anticipated prognostic value for non-RTW: the Numeric Pain Rating Scale (NRS, 9–10 out of a maximum of 10), the Step Test, the Pseudo Strength Test (precipitous cessation, described in the original article) and Behavioural Signs, originally described by Waddel et al. [42]. The best prediction of non-RTW was obtained when at least two out of the four tests were positive (positive predictive value of 0.97 and sensitivity of 0.45).

Loisel et al. [41] studied the Quebec Task Force Classification (QTFC) [43], which classifies patients with LBP based on simple clinical criteria, including signs and symptoms (pain and neurologic examination data), imaging test results and response to treatment. It was designed for several purposes: making clinical decisions, determining prognoses and evaluating quality of care [43]. Subjects classified as having distal radiating pain at baseline (QTFC categories 3 and 4) were likelier to have lower functional status, higher pain level and no return to regular work at the one-year follow-up. The medical history and physical examination allowed the physician to classify the subjects according to the first four categories of the QTFC: QTFC 1 (pain without radiation), QTFC 2 (pain with proximal radiation, i.e., above the knee), QTFC 3 (pain with distal radiation, i.e., below the knee) and QTFC 4 (pain with distal radiation and neurologic signs). The patients with distal radiating pain were also likelier to accumulate more days of full compensation and to have higher treatment costs after a mean follow-up period of 6.5 years.

## 4. Discussion

The literature research yielded seven original articles which were all at least 10 years old. This means that work ability, functional capacity and clinical signs have no longer been the focus of research. In one systematic review [8], it was even recommended to avoid RTW as an outcome measure in studies about chronic LBP. RTW has been considered too complicated as an outcome measure, which may also reflect general attitudes in this research field. A more recent review by Froud et al. [11] found that the number of published LBP trials has increased by a factor of 4.5 per year from 5.4 (1980–1999) to 22.4 (2000–2012). The most common outcome measures were the VAS and the Roland–Morris Disability Questionnaire for functional disability. The authors did not discuss the role of functional measurements at all but focused on the forms and questionnaires. This tallies with our observations.

This poses the question of whether functional tests are outdated. However, functional tests should not be totally ignored. They can have an important impact on medico-legal issues. Methodological problems are obvious when considering the problems relating to the functional tests of patients with LBP. However, if these are totally replaced by patients´ perceptions of pain and disability, the results may be misleading for medical personnel and patients. The concrete measure illustrates important aspects of illness.

When selecting the clinical tests, we omitted the methods requiring special techniques, such as isokinetic measurements [15] or tests developed for an exceptional group such as fire-fighters [44]. The Isernhagen work system was also ignored. Although it proved reliable in functional capacity evaluation in patients with chronic LBP [45,46], it is time-consuming to carry out and requires special arrangements.

We wanted to find tests that would be readily usable and easily available for the physician, physiotherapist or occupational therapist, so that the tests could be recommended for clinical practice. A good test must be reliable and responsive, and it must predict RTW. We understand that functional tests or clinical findings cannot solely be used in the evaluation process of working capacity. Psychosocial factors and work measures play a key role in the evaluation process, but this study focused on physical factors.

Hanke et al. [47] conducted a narrative review of function-based tests to determine the return-to-activity state with non-specific LBP. They identified 33 different tests for which positive statements regarding reliability, validity and relevance for the assessment of return-to-activity status in non-specific back pain could be made. The ability to walk, behaviour when lifting and carrying objects, motor control, muscle strength and mobility play a particular role, according to their study. In our study, we found only seven articles that considered physical performance measurements in RTW evaluation. Hanke et al. [47] included cross-sectional studies, which increased the number of tests. In our study, we required predictive evidence of the test for RTW as a criterion.

In our study, we found four fair and three poor original studies fulfilling our criteria. Three studies classified as fair overlap [36,37,38], because Strand et al. [36] presented the Pick-up test first in 2001 and a year later the BPS, including the Pick-up test as part of the whole BPS [37]. Magnussen et al. [38] used the BPS in their rehabilitation study. However, they stated that evaluation of physical functioning based on physical performance tests was not the focus of their study, and the number of participants completing the programme was small [38]. This is why the evaluation of the BPS is based on Strand et al.’s 2002 research [37]. The advantage of the BPS is that it does not require any special arrangements or techniques, and the reference values of the BPS for healthy persons have been published [48]. Overall, the practical approach of the BPS for functional capacity evaluation seems possible, but further research in different patient samples is needed to clarify the role of BPS in different subjects (different age groups, severity of symptoms, cohort studies and controlled trials of CLBP rehabilitation).

A simple test of back muscle endurance was connected to work capacity in a study by Bendix et al. [39]. Poor back muscle endurance was connected to receipt of a disability pension in their study of a functional restoration programme and the relief of back pain in the functional restoration programme was related to good pre-treatment back muscle endurance. The extent to which this test provokes further back pain has not been reported.

The back endurance test as described by Biering-Sørensen (1984) [49] is “measuring how many seconds the subject is able to keep the unsupported upper body (from the upper border of the iliac crest) horizontal, while placed prone with the buttocks and legs fixed to the couch by three wide canvas straps and the arms folded across the chest”. This is the original test procedure reported by Sorensen and the reference values reported by Alaranta et al. (1994) [50] are based on this. One author’s experience (HH) has been that the test sometimes exacerbates pain. Therefore, careful guidance is needed if this test is used, and one should avoid encouraging the patient too much to avoid muscle strain. The test has proved reliable, is easy to perform and it has published reference values [50].

Kool et al. [40] investigated four tests with an anticipated prognostic value for non-RTW: the Numeric Pain Rating Scale (NRS, 9–10 of a maximum of 10), the Step Test, the Pseudo Strength Test and Behavioural Signs, originally described by Waddel et al. [42]. The predictive value of this test combination is particularly good for non-RTW. Only patients who were willing to return to full-time work were recruited into the study. This is not always the clinical situation when work capacity evaluations are made, which may partly explain the good predictive value of these four tests. However, the practical implications of these observations remain low for the clinician. These tests predict non-RTW, which has a negative connotation. Based on these tests, one cannot deny rehabilitation for a person whose work capacity is jeopardised. In addition, the phenomenon of non-organic signs is obscure. According to Main and Waddell [51], positive behavioural signs should be understood as a response to examination affected by fear in the context of recovery from injury and the development of chronic incapacity. Replication and further development of this test combination is warranted before it can be recommended.

Loisel et al. [41] studied the Quebec Task Force Classification (QTFC) [43], which classifies patients with LBP based on simple clinical criteria, including signs and symptoms (pain and neurologic examination data), imaging test results and response to treatment [43]. Subjects classified as having distal radiating pain at baseline were likelier to have a lower functional status, higher pain level and not to have returned to regular work at the one-year follow-up. For our review, it is interesting that pain radiation is an important indicator and that part of the QTFC can be performed without any demanding imaging technique. This piece of information can be easily gathered from patients as a part of patient history and a part of clinical examination of the patient.

Based on our review, we found the BPS and the back endurance test to be the most promising tests for occupational health service and the clinical practitioner. They are sufficiently simple and reliable, they have reference values and they do not require special equipment, thus keeping the cost of testing low. Most importantly, these tests have relevance in terms of RTW. It would be useful both to replicate the earlier studies and to study them in different working cultures. In the future, one would like to see studies with motor and movement control of LBP patients as an indicator of RTW. A new point of view for future research is provided by smartphone applications in the registration of body movement and activities [52]. To date, studies have concentrated on pain perception as an outcome measure [53,54]. In addition, functional tests could complete the widely used working ability evaluations methods such as the Work Ability Index (WAI) [55]. This calls for new research and development projects.

According to Nguyen et al. [56], as many as 90 percent of persons with occupational nonspecific low back pain are able to return to work in a relatively short period of time as long as no serious conditions relating to LBP, so-called “red flags”, exist. The functional tests can provide further insight into the clinical examination and contribute to the treatment and rehabilitation plan for those 10% of back pain patients who have challenges in RTW.

A limitation of the present study is that we may have missed relevant articles in our search. In addition, we focused only on tests that are applicable by clinical practitioners, and more demanding evaluation protocols have been ignored. We purposely focused on clinical examination and easily available tests, because that is what much clinical decision-making and many medico-legal issues are based on. Special situations such as physically demanding work were outside the scope of this review. Another limiting factor is that studies were heterogeneous in terms of the work situation and the readiness of participants to RTW varied. Clearly, new prospective cohort studies and RCTs are needed in this field, paying special attention to possible sources of bias in the study design.

## 5. Conclusions

We found the BPS and back endurance test to be the most promising tests for patients with LBP, contributing to the evaluation of RTW, but further confirmatory studies are recommended. Radiation of back pain, with or without neurological deficiencies, had some predictive value in terms of RTW. However, evaluating when patients with LBP can resume everyday activities and work without risking recurrence or chronicity is not possible with functional tests alone, and psychosocial aspects and work demands must be considered.

## Figures and Tables

**Table 1:** Definitions of terms.

Concept	Definition of Concept/Reference
Working capacity	Work capacity is the ability to perform real physical work [16].
Working ability	Work ability is the result of the interaction of the worker with his or her work and indicates how good a worker is at present and in the near future, and how able he or she is to do his or her work with respect to work demands, health and mental resources [16].
Work Ability Index (WAI)	The Work Ability Index (WAI) contains questions concerning your work, your work ability and your health. Your answers help to indicate whether measures for improving your health have to be taken and if your work ability must be improved [17].
Low back pain (LBP)	It is defined by the location of pain, typically between the lower rib margins and the buttock creases. It is commonly accompanied by pain in one or both legs and some people with low back pain have associated neurological symptoms in the lower limbs [18].
Chronic low back pain	Chronic low back pain (CLBP) is defined as pain lasting longer than 3 months [7].
Unspecific low back pain	Up to 90% of all LBP instances are considered “un-specific” [4], i.e., the cause of pain is not known [5].
Return to work (RTW)	Return to work (RTW) is a key pillar in a set of workplace processes designed to facilitate the workplace reintegration of persons concerned who experience a reduction in work capacity as a result of either occupational or non-occupational diseases or injuries. The return to work of workers who are on sick leave is part of a continuum of processes aimed at protecting and promoting the health, well-being and work ability of the workforce. Return to work is one important component of a tertiary prevention approach [19].
Physical function/performance	Is defined as one’s ability to carry out activities that require physical actions, ranging from self-care (activities of daily living) to more complex activities that require a combination of skills, often with a social component or within a social context [20].
Functional tests	Functional performance testing means using a set of tests to determine performance abilities or functional limitations. A functional limitation is the inability to perform a particular activity at a normal level [21].

**Table 3:** Outcome of the included articles.

Author, Year, [Reference Number], country	Outcome Measures	Results	Conclusions
Christiansen D et al., 2010 [34], Denmark	Outcomes were obtained by national register data, medical records, and a postal questionnaire	The prognostic value of pain response classification seems limited in patients sick-listed from work because of low back pain (LBP)	The prognostic value of pain response classification seems limited in patients sick-listed from work because of LBP.Back muscle endurance is connected to less dependency on disability pension and reduction in back pain level during 1 year.
Strand LI et al., 2001 [36], Norway	Intertester reliability, validity study, responsiveness study	Reliability: Kappa = 0.74 Validity: Pick-up test showed to be applicable primarily in patients with back pain. Scores on the pick-up test at baseline did not predict return to work at follow-up. Responsiveness: patients who had returned to work after 1 year had improved significantly more on pick-up test scores from baseline to follow-up than patients who had not return to work.	The Pick-up test demonstrated the possession of measurement properties with respect to reliability, validity and responsiveness to change in patients with long-lasting back pain. The physical performance test discriminated between groups of patients with musculoskeletal pain, and showed the highest relevance to patients with back pain. Demographic variables had little influence on test scores. Predictive validity was not demonstrated. The test was responsive to functional change over time. The Pick-up test seems useful as a functional assessment tool and as an outcome tool in patients with back pain. However, the test only assesses one activity, and it is not known if the test reflects the performance of other daily activities requiring dynamic flexibility of the trunk
Strand LI et al., 2002 [37], Norway	RTW	Back Performance Scale (BPS) sum scores discriminated between patients with different RTW status and were higher for back pain than for other musculoskeletal pain. BPS sum was more responsive than the separate tests. BPS is practical measure of performance, being easy and quick to perform, with no need for costly equipment.	The BPS, including 5 physical performance tests of daily activities, appears to be a useful instrument for reflecting key aspects of performance in patients with long-lasting back problems. Internal consistency of the BPS was high, and discriminative ability of the instrument and responsiveness to change were demonstrated. The BPS was shown to be more responsive to change than each of the 5 tests separately. As performance of the 5 tests primarily requires mobility of the trunk in the sagittal plane, the authors believe future research should examine whether tests using side bending and twisting also should be included or could replace other tests to have an even better measure of mobility-related activities in people with back pain. The BPS is a practical measure of performance, being easy and quick to perform, with no need for costly equipment. Reliability of the BPS sum score needs to be established.
Magnussen L et al., 2007 [38], Norway	* RTW or having entered a RTW process * Life satisfaction, disability, fear avoidance behaviour and expectancy	Intervention had no statistical significant effect on RTW The disability pensioners who at baseline had positive expectancy, less pain and better physical performance were most likely to have entered a RTW process at follow-up.	Positive expectancy, better physical performance and less pain were related to RTW.
Bendix AF et al., 1998 [39], Denmark	* Ability to work * Disability pension obtained or application pending * Completion versus withdrawal from treatment * Change in back and leg pain * Change in level of activities of daily living * Subjective overall assessment of back problems	* Young age correlates positively to return to work (RTW) * Women were almost twice as likely to return to work than men (gender) * There was a positive correlation with high severity of leg pain and low back muscle endurance vs. pension obtained or application pending in the functional restoration (FR) group	* Different factors can be identified as predictive of outcome in a functional restoration program, but most of these factors were also shown to predict success for shorter control outpatient programs or of no treatment. * Of the physical findings, pretreatment back muscle endurance was the most important factor and it was positively correlated with less dependency on disability pension and reduction of back pain level during one year
Kool J et al., 2002 [40], Switzerland	The study investigated four tests with an anticipated prognostic value for non-RTW: the Numeric Pain Rating Scale (NRS, 9–10 of a maximum of 10), the Step Test and Pseudo Strength Test and Behavioural SignsThe RTW rate was obtained for sick-listing by postal surveys	A very good positive predictive value of 0.97 with a sensitivity of 0.45 is achieved by the combination of these three (Step Test, Pseudo Strength Test, Pain rating 9 or 10) tests with the Behavioural Signs. These are interpreted as positive when two or more of these four tests are positive.	The combination of the four prognostic tests allows a reliable prognosis of non-RTW. Unemployment, time off work, nationality and physicalwork load were less predictive.
Loisel P et al., 2002 [41], Canada	Functional status, pain level and work status were assessed at baseline and after one year: * Generic Sickness Impact Profile (SIP) * Oswestry Questionnaire * McGill Pain Questionnaire	* Subjects classified as having distal radiating pain (categories 3 and 4) at baseline were more likely to have a lower functional status, higher pain level, and no return to regular work at the 1-year follow-up evaluation. They also were more likely to accumulate more days of full compensation and to cost more after a mean follow-up period of 6.5 years.	The Quebec Task Force Classification (QTFC) demonstrated good predictive ability by discriminating between subjects with and those without distal radiating pain. This discimination had impact on the prognosis of LBP and RTW.

FR = Functional Restoration; LBP = low back pain; RTW = return to work; QTFC = Quebec Task Force Classification; BPS = Back Performance Scale.

## Data Availability

Not applicable.

## References

[1] Vlaeyen J.W.S., Maher C.G., Wiech K., Van Zundert J., Meloto C.B., Diatchenko L., Battie M.C., Goossens M., Koes B., Linton S.J. (2018). Low back pain. Nat. Rev. Dis. Prim..

[2] Wu A., March L., Zheng X., Huang J., Wang X., Zhao J., Blyth F.M., Smith E., Buchbinder R., Hoy D. (2020). Global low back pain prevalence and years lived with disability from 1990 to 2017: Estimates from the Global Burden of Disease Study 2017. Ann. Transl. Med..

[3] Von Korff M., Crane P., Lane M., Miglioretti D.L., Simon G., Saunders K., Stang P., Brandenburg N., Kessler R. (2005). Chronic spinal pain and physical-mental comorbidity in the United States: Results from the national comorbidity survey replication. Pain.

[4] Oliveira C.B., Maher C.G., Pinto R.Z., Traeger A.C., Lin C.C., Chenot J.F., van Tulder M., Koes B.W. (2018). Clinical practice guidelines for the management of non-specific low back pain in primary care: An updated overview. Eur. Spine J..

[5] Maher C., Underwood M., Buchbinder R. (2017). Non-specific low back pain. Lancet.

[6] Meucci R.D., Fassa A.G., Faria N.M. (2015). Prevalence of chronic low back pain: Systematic review. Rev. Saude Publica.

[7] Urits I., Burshtein A., Sharma M., Testa L., Gold P.A., Orhurhu V., Viswanath O., Jones M.R., Sidransky M.A., Spektor B. (2019). Low Back Pain, a Comprehensive Review: Pathophysiology, Diagnosis, and Treatment. Curr. Pain Headache Rep..

[8] Chapman J.R., Norvell D.C., Hermsmeyer J.T., Bransford R.J., Devine J., McGirt M.J., Lee M.J. (2011). Evaluating common outcomes for measuring treatment success for chronic low back pain. Spine.

[9] Pekkanen L., Kautiainen H., Ylinen J., Salo P., Hakkinen A. (2011). reliability and validity study of the Finnish version 2.0 of the Oswestry Disability Index. Spine.

[10] Brouwer S., Kuijer W., Dijkstra P.U., Göeken L.N., Groothoff J.W., Geertzen J.H. (2004). Reliability and stability of the Roland Morris Disability Questionnaire: Intra class correlation and limits of agreement. Disabil. Rehabil..

[11] Froud R., Patel S., Rajendran D., Bright P., Bjørkli T., Buchbinder R., Eldridge S., Underwood M. (2016). A systematic review of outcome measures use, analytical approaches, reporting methods, and publication volume by year in low back pain trials published between 1980 and 2012: Respice, adspice, et prospice. PLoS ONE.

[12] World Health Organization (2001). International Classification of Functioning, Disability and Health: ICF.

[13] Mattila-Holappa P., Kausto J., Aalto V., Kaila-Kangas L., Kivimäki M., Oksanen T., Ervasti J. (2021). Alternative duty work as workplace-initiated procedure to reduce sickness absence. BMC Public Health.

[14] Spreitzer G., Cameron L., Garrett L. (2017). Alternative work arrangements: Two images of the new world of work. Annu. Rev. Organ. Psychol. Organ. Behav..

[15] Rothstein J.M., Lamb R.L., Mayhew T.P. (1987). Clinical uses of isokinetic measurements. Critical issues. Phys. Ther..

[16] Anbazhagan S., Ramesh N., Surekha A., Fathima F.N., Melina A. (2016). Estimation of work capacity and work ability among plantation workers in South India. Indian J. Occup. Environ. Med..

[17] Tuomi K., Ilmarinen J., Jahkola A., Katajarinne L., Tulkki A. (1998). Work Ability Index.

[18] Hartvigsen J., Hancock M.J., Kongsted A., Louw Q., Ferreira M.L., Genevay S., Hoy D., Karppinen J., Pransky G., Sieper J. (2018). Lancet Low Back Pain Series Working Group. What low back pain is and why we need to pay attention. Lancet.

[19] International Social Security Association. https://ww1.issa.int/guidelines/rtw/174799.

[20] Van Lummel R.C., Walgaard S., Pijnappels M., Elders P.J., Garcia-Aymerich J., van Dieën J.H., Beek P.J. (2015). Physical Performance and Physical Activity in Older Adults: Associated but Separate Domains of Physical Function in Old Age. PLoS ONE.

[21] Reiman M.P., Manske R.C. What Is Functional Testing?. https://us.humankinetics.com/blogs/excerpt/what-is-functional-testing.

[22] Page M.J., McKenzie J.E., Bossuyt P.M., Boutron I., Hoffmann T.C., Mulrow C.D., Shamseer L., Tetzlaff J.M., Akl E.A., Brennan S.E. (2021). The PRISMA 2020 statement: An updated guideline for reporting systematic reviews. BMJ.

[23] National Heart, Lung, and Blood Institute Study Quality Assessment Tool. https://www.nhlbi.nih.gov/health-topics/study-quality-assessment-tools.

[24] Furlan A.D., Pennick V., Bombardier C., van Tulder M., Editorial Board, Cochrane Back Review Group (2009). 2009 updated method guidelines for systematic reviews in the Cochrane Back Review Group. Spine.

[25] Dubourg G., Rozenberg S., Fautrel B., Valls-Bellec I., Bissery A., Lang T., Faillot T., Duplan B., Briançon D., Levy-Weil F. (2002). A pilot study on the recovery from paresis after lumbar disc herniation. Spine.

[26] Ferguson S.A., Marras W.S., Gupta P. (2000). Longitudinal quantitative measures of the natural course of low back pain recovery. Spine.

[27] Balagué F., Nordin M., Sheikhzadeh A., Echegoyen A.-C., Brisby H., Hoogewoud H.M., Fredman P., Skovron M.L. (1999). Recovery of severe sciatica. Spine.

[28] Parks K.A., Crichton K.S., Goldford R.J., McGill S.M. (2003). A comparison of lumbar range of motion and functional ability scores in patients with low back pain: Assessment for range of motion validity. Spine.

[29] Du Bois M., Szpalski M., Donceel P. (2009). Patients at risk for long-term sick leave because of low back pain. Spine J..

[30] Bruno P.A., Goertzen D.A., Millar D.P. (2014). Patient-reported perception of difficulty as a clinical indicator of dysfunctional neuromuscular control during the prone hip extension test and active straight leg raise test. Man. Ther..

[31] Davis A.M., Bridge P., Miller J., Nelson-Wong E. (2011). Interrater and intrarater reliability of the active hip abduction test. J. Orthop. Sports Phys. Ther..

[32] Farasyn A., Meeusen R., Nijs J., Cuesta-Vargas A. (2013). Exploration of the validity and reliability of the “backache disability index” (BADIX) in patients with non-specific low back pain. J. Back Musculoskelet. Rehabil..

[33] Kamper S.J., Stanton T.R., Williams C.M., Maher C.G., Hush J.M. (2011). How is recovery from low back pain measured? A systematic review of the literature. Eur. Spine J..

[34] Christiansen D., Larsen K., Jensen O.K., Nielsen C.V. (2010). Pain response classification does not predict long-term outcome in patients with low back pain who are sick-listed. J. Orthop. Sports Phys. Ther..

[35] McKenzie R., May S. (2003). The Lumbar Spine: Mechanical Diagnosis & Therapy. Vol. 2.

[36] Strand L.I., Ljunggren A.E. (2001). The pick-up test for assessing performance of a daily activity in patients with back pain. Adv. Ther..

[37] Strand L.I., Moe-Nilssen R., Ljunggren A.E. (2002). Back performance scale for the assessment of mobility-related activities in people with back pain. Phys. Ther..

[38] Magnussen L., Strand L.I., Skouen J.S., Eriksen H.R. (2007). Motivating disability pensioners with back pain to return to work--a randomized controlled trial. J. Rehabil. Med..

[39] Bendix A.F., Bendix T., Haestrup C. (1998). Can it be predicted which patients with chronic low back pain should be offered tertiary rehabilitation in a functional restoration program? A search for demographic, socioeconomic, and physical predictors. Spine.

[40] Kool J.P., Oesch P.R., de Bie R.A. (2002). Predictive tests for non-return to work in patients with chronic low back pain. Eur. Spine J..

[41] Loisel P., Vachon B., Lemaire J., Durand M.-J., Poitras S., Stock S., Tremblay C. (2002). Discriminative and predictive validity assessment of the Quebec Task Force classification. Spine.

[42] Waddell G., McCulloch J.A., Kummel E., Venner R.M. (1980). Nonorganic physical signs in low-back pain. Spine.

[43] Spitzer W.O. (1987). Scientific approach to the assessment and management of activity-related spinal disorders. A monograph for clinicians. Report of the Quebec Task Force on Spinal Disorders. Spine.

[44] Lusa S., Halonen J., Punakallio A., Wikström M., Lindholm H., Luukkonen R. (2015). Pelastajien Fyysisen Toimintakyvyn ArviointijärJestelmän Käytettävyys ja FireFit-Indeksin Kehittäminen: FireFit-Hankkeen IV Vaihe.

[45] Brouwer S., Reneman M.F., Dijkstra P.U., Groothoff J.W., Schellekens J.M., Goeken L.N. (2003). Test-retest reliability of the Isernhagen Work Systems Functional Capacity Evaluation in patients with chronic low back pain. J. Occup. Rehabil..

[46] De Baets S., Calders P., Schalley N., Vermeulen K., Vertriest S., Van Peteghem L., Coussens M., Malfait F., Vanderstraeten G., Van Hove G. (2018). Updating the evidence on functional capacity evaluation methods: A systematic review. J. Occup. Rehabil..

[47] Hanke A., Schoch W., Keller M., Kurz E., Richter R. (2022). Funktionelle Testungen zur Ermittlung des Return-to-Activity-Status bei Patienten mit unspezifischen Kreuzschmerzen. Sport. Sportschaden.

[48] Strand L.I. (2017). Back Performance Scale. J. Physiother..

[49] Biering–Sørensen F. (1984). Physical measurements as risk indicators for low-back trouble over a one-year period. Spine.

[50] Alaranta H., Hurri H., Heliovaara M., Soukka A., Harju R. (1994). Non-dynamometric trunk performance tests: Reliability and normative data. Scand. J. Rehabil. Med..

[51] Main C.J., Waddell G. (1998). Behavioral responses to examination. A reappraisal of the interpretation of “nonorganic signs”. Spine.

[52] Elgueta-Cancino E., Rice K., Abichandani D., Falla D. (2022). Measurement properties of smartphone applications for the measurement of neck range of motion: A systematic review and meta analyses. BMC Musculoskelet. Disord..

[53] Bagg M.K., Wand B.M., Cashin A.G., Lee H., Hubscher M., Stanton T.R., O’Connell N.E., O’Hagan E.T., Rizzo R.R.N., Wewege M.A. (2022). Effect of graded sensory retraining on pain intensity in patients with chronic low back pain: A randomized clinical trial. JAMA.

[54] Takala E.P., Viikari-Juntura E. (2000). Do functional tests predict low back pain?. Spine.

[55] European Aviation Group for Occupational Safety and Health (EAGOSH). http://www.eagosh.org/eagosh-files/EAGOSH_WAI.pdf.

[56] Nguyen T.H., Randolph D.C. (2007). Nonspecific low back pain and return to work. Am. Fam. Phys..

